# Computational Applications in Secondary Metabolite Discovery (CAiSMD): an online workshop

**DOI:** 10.1186/s13321-021-00546-8

**Published:** 2021-09-06

**Authors:** Fidele Ntie-Kang, Kiran K. Telukunta, Serge A. T. Fobofou, Victor Chukwudi Osamor, Samuel A. Egieyeh, Marilia Valli, Yannick Djoumbou-Feunang, Maria Sorokina, Conrad Stork, Neann Mathai, Paul Zierep, Ana L. Chávez-Hernández, Miquel Duran-Frigola, Smith B. Babiaka, Romuald Tematio Fouedjou, Donatus B. Eni, Simeon Akame, Augustine B. Arreyetta-Bawak, Oyere T. Ebob, Jonathan A. Metuge, Boris D. Bekono, Mustafa A. Isa, Raphael Onuku, Daniel M. Shadrack, Thommas M. Musyoka, Vaishali M. Patil, Justin J. J. van der Hooft, Vanderlan da Silva Bolzani, José L. Medina-Franco, Johannes Kirchmair, Tilmann Weber, Özlem Tastan Bishop, Marnix H. Medema, Ludger A. Wessjohann, Jutta Ludwig-Müller

**Affiliations:** 1 Department of Chemistry, University of Buea, P. O. Box 63, Buea, Cameroon; 2 Institute of Pharmacy, Martin-Luther University of Halle-Wittenberg, Kurt-Mothes-Str. 3, 06120 Halle, Germany; 3 Institute of Botany, Technische Universität Dresden, Zellescher Weg 20b, 01062 Dresden, Germany; 4 Tarunavadaanenasaha Muktbharatonnayana Samstha Foundation, Hyderabad, India; 5 Institute of Pharmaceutical Biology, Technische Universität Braunschweig, Mendelssohnstrasse 1, 38106 Braunschweig, Germany; 6 Department of Computer and Information Sciences, Colege of Science and Technology, Covenant University, Km. 10 Idiroko Rd, Ogun Ota, Nigeria; 7 School of Pharmacy, University of the Western Cape, Cape Town, 7535 South Africa; 8 South African Medical Research Council Bioinformatics Unit, South African National Bioinformatics Institute, University of the Western Cape, Cape Town, 7535 South Africa; 9 Nuclei of Bioassays, Biosynthesis and Ecophysiology of Natural Products (NuBBE), Department of Organic Chemistry, Institute of Chemistry, Sao Paulo State University–UNESP, Araraquara, Brazil; 10 Corteva Agriscience, Indianapolis, IN USA; 11 Institute for Inorganic and Analytical Chemistry, Friedrich Schiller University, Jena, Germany; 12 Center for Bioinformatics, Universität Hamburg, 20146 Hamburg, Germany; 13 Department of Chemistry and Computational Biology Unit (CBU), University of Bergen, 5020 Bergen, Norway; 14 Pharmaceutical Bioinformatics, Albert-Ludwigs-University, Freiburg, Germany; 15 DIFACQUIM Research Group, Department of Pharmacy, School of Chemistry, Universidad Nacional Autónoma de México, Mexico City, Mexico; 16 Ersilia Open Source Initiative, Cambridge, UK; 17 Joint IRB-BSC-CRG Programme in Computational Biology, Institute for Research in Biomedicine (IRB Barcelona), The Barcelona Institute of Science and Technology, Barcelona, Catalonia Spain; 18 Department of Chemistry, University of Dschang, Dschang, Cameroon; 19 Department of Immunology, School of Health Sciences, Catholic University of Central Africa, BP 7871, Yaoundé, Cameroon; 20 Department of Biochemistry and Molecular Biology, University of Buea, P. O. Box 63, Buea, Cameroon; 21 Department of Physics, Ecole Normale Supérieure, University of Yaoundé I, BP. 47, Yaoundé, Cameroon; 22 Bioinformatics and Computational Biology Lab, Department of Microbiology, Faculty of Sciences, University of Maiduguri, P.M.B. 1069, Maiduguri, Borno State Nigeria; 23 Department of Pharmaceutical and Medicinal Chemistry, Faculty of Pharmaceutical Sciences, University of Nigeria Nsukka, Nsukka, Nigeria; 24 Department of Chemistry, St. John’s University of Tanzania, P. O. Box 47, Dodoma, Tanzania; 25 Research Unit in Bioinformatics (RUBi), Department of Biochemistry and Microbiology, Rhodes University, Makhanda, 6140 South Africa; 26 Computer Aided Drug Design Lab, KIET Group of Institutions, Delhi-NCR, Ghaziabad, 201206 India; 27 Bioinformatics Group, Wageningen University, Wageningen, The Netherlands; 28 Department of Pharmaceutical Sciences, Division of Pharmaceutical Chemistry, University of Vienna, 1090 Vienna, Austria; 29 The Novo Nordisk Foundation Center for Biosustainability, Technical University of Denmark, Kgs. Lyngby, Denmark; 30 Department of Bioorganic Chemistry, Leibniz Institute of Plant Biochemistry (IPB), Weinberg 3, 06120 Halle (Saale), Germany; 31 German Centre for Integrative Biodiversity Research (iDiv), Puschstraße 4, 04103 Leipzig, Germany

**Keywords:** Bioinformatics, Chemoinformatics, Metabolites, Online workshop, Predictions, Web tools

## Abstract

We report the major conclusions of the online open-access workshop “Computational Applications in Secondary Metabolite Discovery (CAiSMD)” that took place from 08 to 10 March 2021. Invited speakers from academia and industry and about 200 registered participants from five continents (Africa, Asia, Europe, South America, and North America) took part in the workshop. The workshop highlighted the potential applications of computational methodologies in the search for secondary metabolites (SMs) or natural products (NPs) as potential drugs and drug leads. During 3 days, the participants of this online workshop received an overview of modern computer-based approaches for exploring NP discovery in the “omics” age. The invited experts gave keynote lectures, trained participants in hands-on sessions, and held round table discussions. This was followed by oral presentations with much interaction between the speakers and the audience. Selected applicants (early-career scientists) were offered the opportunity to give oral presentations (15 min) and present posters in the form of flash presentations (5 min) upon submission of an abstract. The final program available on the workshop website (https://caismd.indiayouth.info/) comprised of 4 keynote lectures (KLs), 12 oral presentations (OPs), 2 round table discussions (RTDs), and 5 hands-on sessions (HSs). This meeting report also references internet resources for computational biology in the area of secondary metabolites that are of use outside of the workshop areas and will constitute a long-term valuable source for the community. The workshop concluded with an online survey form to be completed by speakers and participants for the goal of improving any subsequent editions.

**Supplementary Information:**

The online version contains supplementary material available at 10.1186/s13321-021-00546-8.

## Introduction

Natural products (NPs) have potential therapeutic uses, either directly as drugs or as lead compounds [1]. The discovery of secondary metabolites (SMs) from bacteria, fungi, and plants as lead compounds for drug discovery purposes by pharmaceutical companies had been slowed down before the last decade, despite their huge representation among compounds approved as drugs. For example, in the area of cancer drug discovery, during the period 1946–1980, 40 out of the 75 approved small molecules by the United States Food and Drug Administration (FDA) were with NPs or NP-derived [2]. During the past decade, SM discovery has been enhanced by the rapid progress in artificial intelligence and its applications [3]. Research in the field of NPs has therefore embraced the need for large-scale analysis of digitized experimental data in the fields of metabolomics, transcriptomics, genomics, often referred to as the “omics” era [4]. This calls for the need for NP chemists to be properly trained in the new “omics” disciplines to be able to tackle the new challenges in the identification of SM, elucidation of their structures, modes of action, and potential toxicities in order to enhance drug discovery from nature.

With the advent of the COVID-19 lockdown associated with travel restrictions and social distancing measures, scientists had to resort to training and sharing of research results via the web [5]. Although online teaching may prove challenging in the sense that it is often difficult to ascertain the level of concentration of the learners and it is not possible to get their immediate response from facial gestures, distance learning has proven to be one of the feasible approaches to ensure that teaching and learning still continue in the midst of the pandemic [6, 7]. Most institutions (mostly in secondary and tertiary education) have resorted to online teaching, while some are still battling with maintaining a minimal amount of face-to-face teaching. This is often for social reasons and to ensure that the educators and learners get feedback from the past lessons and have the possibility to correct exercises and get responses from pressing issues and misunderstood concepts. As a result, modern tools to enhance learning while maintaining barrier measures are in high demand and web conferences have almost completely replaced the traditional scientific conferences and workshops, which have now undergone some hibernation to slow down the spread of the virus [8, 9]. Within the context of the German Academic Exchange Services (DAAD) funding scheme, invited scholars from abroad are encouraged to organize a training event, which could be in the form of a digitalized lecture (i.e. workshops, conferences, course tutorials, etc.) (https://www.daad.org/en/find-funding/faculty/visiting-professorship/). It is within this context that we proposed to organize the online workshop entitled “Computational Applications in Secondary Metabolite Discovery” (CAiSMD).

This virtual workshop introduced participants to modern computer-based approaches and tools for the exploration of the NPs and “omics” world. Most of the tools (software, web servers, databases, etc.), methods and results presented to the participants were recent (dating from 2019 and later). The focus was on bioinformatics, chemoinformatics, NP chemistry, computational drug design, and genomic analysis, with applications in drug discovery. The organizers took the initiative to start identifying, inviting, and corresponding with key experts in the field who could provide inputs in the form of keynote lectures, oral presentations and online hands-on training tutorials. All information regarding deadlines and registration were published on the workshop website from which interested applicants could download an abstract template and make all relevant uploads. The cost-free workshop was conducted in English and open to the entire scientific community. M.Sc. and Ph.D. students, postdoctoral researchers and early-career scientists were the target group of the workshop. Selected participants could submit an abstract indicating if they wish to give 15-min oral presentations.

All sessions with oral presentations and the parallel hands-on sessions (HS) were accessible through Zoom. All digital references are summarized in Table 1 and the final program and hand-out sessions are available on the web site (https://caismd.indiayouth.info/). Since the workshop was intended in large part to attract early-career researchers, two formats were included to attract them: parallel hands-on sessions and round table discussions, partially led by early-career post-docs (Additional files 1, 2).

**Table 1:** Important web links shared during the CAiSMD workshop

Speaker (lecture)	Group website (web link to tools presented)	References
Ludger A. Wessjohann (KL01)	Research group: http://www.ipb-halle.de/en/research/bioorganic-chemistry/	
Email: wessjohann@ipb-halle.de	Tools: Databases and Tools, e.g. for computational mass spectrometry (https://www.ipb-halle.de/en/infrastructure/databases-and-tools/?MP=6-1058)	[48–50]
Marnix Medema (KL02)	Research group: http://marnixmedema.nl/	
Email: marnix.medema@wur.nl	Tools: the antiSMASH software (https://antismash.secondarymetabolites.org); the antiSMASH database (https://antismash-db.secondarymetabolites.org/); Paired Omics Data Platform (https://pairedomicsdata.bioinformatics.nl); the plantiSMASH software (http://plantismash.secondarymetabolites.org/)	[13–15, 51, 52]
Tilmann Weber (KL03)	Research group: https://www.biosustain.dtu.dk/	
Email: tiwe@biosustain.dtu.dk	Tools: the antiSMASH software (https://antismash.secondarymetabolites.org); the antiSMASH database (https://antismash-db.secondarymetabolites.org/); CRISPy (https://crispy.secondarymetabolites.org); the secondary metabolite bioinformatics portal (http://www.secondarymetabolites.org/)	[14, 15, 52, 53]
ÖzlemTastan Bishop (KL04)	Research group: https://rubi.ru.ac.za/	
Email: o.tastanbishop@ru.ac.za	Tools: SANCDB compound database (https://sancdb.rubi.ru.ac.za/)	[35, 54]
Justin J. J. van der Hooft (HS01)	Research group: https://www.wur.nl/en/Persons/Justin-dr.-JJJ-Justin-van-der-Hooft.htm	
Email: justin.vanderhooft@wur.nl	Tools: GNPS (https://gnps.ucsd.edu/ProteoSAFe/static/gnps-splash.jsp); MS2LDA (http://www.ms2lda.org); MAGMa (https://www.emetabolomics.org/); Paired Omics Data Platform (https://pairedomicsdata.bioinformatics.nl)	[34, 51, 52, 55, 56]
Daniel M Shadrack (HS02)	Research group:	
Email: dmssjut@gmail.com	Tools: none	
Thommas Musyoka (HS02)	Research group: same as KL04	
Email: mutemibiochemistry@gmail.com	Tools: same as KL04	
Fidele Ntie-Kang (HS02)	Research group: https://ntiekfidele.jimdofree.com/	
Email: fidele.ntie-kang@ubuea.cm	Tools: ANPDB compound database (http://african-compounds.org/anpdb/)	[36, 37]
Yannick Djoumbou-Feunang (HS03)	Research group:	
Email: yannick.djoumboufeunang@corteva.com	Tools: BioTransformer (http://biotransformer.ca/)	[26]
Conrad Stork (HS04)	Research group: https://www.zbh.uni-hamburg.de/personen/acm/cstork.html	
Email: stork@zbh.uni-hamburg.de	Tools: NERDD web server (https://nerdd.univie.ac.at/); FAME3 (https://nerdd.univie.ac.at/fame3/); GLORY (https://nerdd.univie.ac.at/glory/); GLORYx (https://nerdd.univie.ac.at/gloryx/); Hit Dexter 2.0 (https://nerdd.univie.ac.at/hitdexter/); NP-Scout (https://nerdd.univie.ac.at/npscout/); Skin Doctor CP (https://nerdd.univie.ac.at/skinDoctorII/)	[17–23]
Neann Mathai (HS04)	Research group: https://www.uib.no/en/persons/Neann.Sarah.Mathai	
Email: neann.mathai@uib.no	Tools: Same as above	
Maria Sorokina (HS05)	Research group: https://cheminf.uni-jena.de/members/maria-sorokina/	
Email: maria.sorokina@uni-jena.de	Tools: CDK (https://cdk.github.io/); COCONUT compound database (https://coconut.naturalproducts.net); NP-likeness score web application (https://naples.naturalproducts.net/); Sugar Removal Utility (https://sugar.naturalproducts.net/)	[38–43]
Kiran K. Telukunta (OP01)	Research group:	
Email: kiran.telukunta@indiayouth.info	Tools: galaxy tutorials (https://galaxyproject.org/learn/)	[57]
Paul F. Zierep (OP02)	Research group: http://www.pharmbioinf.uni-freiburg.de/main/members/ziereppaul/	
Email: paul.zierep@gmail.com	Tools: sempi 2.0 web server (http://sempi.pharmazie.uni-freiburg.de/); StreptomeDB compound database (http://thymin.pharmazie.uni-freiburg.de/streptomedb/); the ANPDB compound database (http://african-compounds.org/anpdb/)	[22, 36, 37, 58]
Victor C. Osamor (OP03)	Research group: https://covenantuniversity.edu.ng/Profiles/Osamor-Victor-Chukwudi#.YGYag1UzbIU	
Email: victor.osamor@covenantuniversity.edu.ng; vcosamor@gmail.com	Tools: OsamorSoft (not available)	[23]
Marilia Valli (OP04)	Research group: https://nubbe.iq.unesp.br/portal/index.html	
Email: mariliava@gmail.com	Tools: NuBBE database (https://nubbe.iq.unesp.br/portal/nubbe-search.html)	[59, 60]
Vanderlan da Silva Bolzani (OP04)	Research group: same as above	
Email: vanderlan.bolzani@unesp.br	Tools: same as above	
Samuel Egieyeh (OP05)	Research group:	
Email: segieyeh@uwc.ac.za	Tools: none	
Miquel Duran-Frigola (OP06)	Research group: https://ersilia.io/	
Email: miquel@ersilia.io	Tools: Chemical Checker (https://pypi.org/project/chemicalchecker/)	[61]
Yannick Djoumbou-Feunang (OP07)	Research group: same as HS03	
Email: yannick.djoumboufeunang@corteva.com	Tools: same as HS03	
Ana L. Chávez-Hernández (OP08)	Research group: https://www.difacquim.com/	
Email: anachavez3026@gmail.com	Tools: Epigenetic Target Profiler web server (http://www.epigenetictargetprofiler.com); BIOFACQUIM natural products database and other tools, e.g. Platform for Unified Molecular Analysis (PUMA), Activity Landscape Plotter, etc. (https://www.difacquim.com/d-tools/)	[62, 63]
José L. Medina-Franco (OP08)	Research group: same as above	
Email: jose.medina.franco@gmail.com	Tools: same as above	
Johannes Kirchmair (OP09)	Research group: https://comp3d.univie.ac.at/the-comp3d-team/johannes-kirchmair/	
Email: johannes.kirchmair@univie.ac.at	Tools: same as HS04	
Maria Sorokina (OP10)	Research group: same as HS05	
Email: maria.sorokina@uni-jena.de	Tools: same as HS05	
Vaishali M. Patil (OP11)	Research group:	
Email: vaishuwise@gmail.com	Tools: none	
Romuald Tematio Fouedjou (OP12)	Research group:	
Email: tematioromy@yahoo.fr	Tools: none	

## Workshop contents

### Keynote lectures

Four keynote lectures (KLs) were given during the workshop, each lasting 45 min. The first KL was held by Ludger Wessjohann, who presented an overview of how various informatics methods and tools developed by his group (along with partners) could support the selection of sources and the identification of NPs of relevance [10–12]. The lecture was focused on the chemoinformatic analysis of:

1. Large databases and text corpuses, and
2. The role of metabolic profiling in future bioactive compound discovery, going beyond classical isolation processes.

An example included in the lecture was a survey of the flora of Java (Indonesia), metabolic profiling of various medicinal plants, with an emphasis on *Hypericum* spp. (St. John’s wort).

At the beginning of day 2, the participants followed the second keynote lecture by Marnix Medema, who highlighted the ongoing work in his research group. His group is developing and using computational methods to identify plant-based, fungal, and bacterial molecules of ecological and clinical importance and developing approaches to assess and predict the biological activities of specialized metabolites to accelerate NP discovery by focusing efforts on the most promising candidates. In his keynote, Medema showed the importance of computational methodologies in the study of microbe-microbe and host-microbe interactions in human, plant and animal microbiomes. Specifically, he discussed the use of computational approaches to investigate biosynthetic diversity across large numbers of genomes, and integrative genome/metabolome mining to link gene clusters to molecules facilitated by novel community-based efforts such as the Paired Omics Data Platform (https://pairedomicsdata.bioinformatics.nl) [13].

The third KL took place at the end of day 2, during the session entitled “looking into the future”, after several oral presentations (OPs). Tilmann Weber presented the latest version (v6) of the antiSMASH genome mining platform (https://antismash.secondarymetabolites.org) [14], which has been under development since 2011, coordinated by his group and that of Marnix Medema. The version 6 of antiSMASH includes an improved user interface, new detection modules, a new cluster comparison tool, and many internal optimizations. These were presented as useful tools for the easy analysis of genomic sequences for the presence of secondary metabolite biosynthetic gene clusters (BGCs) in bacteria and fungi. Additionally, the antiSMASH database (https://antismash-db.secondarymetabolites.org/) [15] was presented as a user-friendly application that allows users to browse and query pre-computed antiSMASH v5 annotations. The database contains information on 147,517 high-quality BGC regions from 388 archaeal, 25,236 bacterial and 177 fungal genomes. It was highlighted that these basic genome mining technologies build the foundations of further in silico studies towards a more comprehensive “Genome Analytics” platform, which could be used to streamline NP discovery and characterization efforts in the future.

The fourth KL was given by Özlem Tastan Bishop on day 3. The presenter showed some early drug discovery research experiences within the context of Africa. The main interests include the identification of novel and alternative drug targeting sites (i.e. allosteric sites) and of hit compounds for communicable and non-communicable diseases, which have recently been published by her group [16–21]. Her group had shown interest to understand the effects of nonsynonymous single nucleotide variations (nsSNVs) on protein structure and function, in order to (i) assess the reasons behind many inherited diseases, (ii) uncover the association to drug resistance mechanisms, and iii) link to drug sensitivity issues in certain populations for precision medicine development; and many other applications. The lecturer also argued that an understanding of the underlying resistance mechanism due to variations at the molecular level is essential and can lead either to modifications of currently approved drugs to get more effective ones or to the design of new inhibitors that overcome resistance mutations. The lecture concluded with recent work on understanding the underlying drug resistance mechanisms and identification of allosteric modulators as an alternative to orthosteric drugs.

### Oral presentations

There were 12 scheduled OPs. The lecture topics included presentations of both novel computational methodologies as well as recent results, e.g., methods for clustering of specialized metabolites and the introduction of a large integrated and open database for NPs (https://coconut.naturalproducts.net) and the most recent version of the NuBBE natural products database from Brazil. Other speakers presented web servers for prediction of metabolites from gene cluster data, e.g., the SeMPI 2.0 web server (http://sempi.pharmazie.uni-freiburg.de/) presented by Paul Zierep for polyketide synthase (PKS) and non-ribosomal peptide synthase (NRPS) prediction by combining with metabolite screening in natural product databases [22] or drug discovery platforms, e.g. that of the University of West Cape (South Africa) presented by Samuel A. Egieyeh. Other methods presented were those implemented in software, e.g. the OsamorSoft spreadsheets, the tool developed in the group of Victor C. Osamor for clustering genomic data [23], with potentially useful applications in plant-based SMs as lead compounds in two databases from African medicinal plants, with a focus on the NANPDB and EANPDB databases (see Table 1).

The afternoon and early evening sessions consisted of 5 core chemoinformatics lectures. In the quest to predict the biological activities of non-characterized NPs, Miquel Duran-Frigola presented the tool Chemical Checker (CC), which included a collection of deep neural networks capable of inferring bioactivity signatures for any compound of interest, even when little or no experimental information is available for them [24]. The speaker also showed how inferred bioactivity signatures are useful to navigate the chemical space in a biologically relevant manner, unveiling higher-order organization in NP collections. The lecturer also used an implementation of a battery of signature-activity relationship models to show that this resulted in a substantial improvement in predictive performance, with respect to chemistry-based classifiers, across a series of biophysics and physiology activity prediction benchmarks [25]. It could, therefore, be concluded that from the CC, large-scale inference of bioactivity profiles can set the basis for automated annotation of compound collections, including drugs, metabolites and NPs.

Yannick Djoumbou-Feunang highlighted how artificial intelligence in the form of machine learning could be useful for secondary metabolite prediction. In silico metabolism prediction tools provide a unique perspective to studying the chemical exposome, and how its changes affect the environment. Classical applications of such tools include, but are not limited to metabolite discovery, environmental fate prediction, ADMET profiling, and molecular design. Several approaches and methods to address the prediction of secondary metabolites have been described, and implemented in a comprehensive list of tools that include expert-, machine learning-, and quantum mechanics (QM)-based systems, or hybrids thereof. However, the speaker showed that in spite of the numerous reported successes, many limitations still hamper the wide adoption of those tools. In his presentation, he described the impact of artificial intelligence in the development of secondary metabolite prediction systems, along with the most commonly implemented approaches. He then showed examples of the application of in silico metabolism prediction tools, such as BioTransformer which he developed [26], in the identification of secondary metabolites. This tool could be useful for identifying the plausible metabolites of a compound, including NPs. He concluded by showing some of the prevalent limitations that hamper the widespread adoption of such tools and propose solutions.

Next, Ana L. Chávez-Hernández, a Ph.D. student from José L. Medina-Franco’s team in Mexico, presented a fragment library of NPs and compound databases for drug discovery, laying emphasis on the fragment libraries were generated from the recently published COlleCtion of Open NatUral producTs (COCONUT) [27] and other reference-data sets such as food chemical compounds (FooDB) [28], including compounds from a focused library from the Chemical Abstract Service (CAS) and inhibitors of the main protease of SARS-CoV-2 (3CLP). The fragment libraries generated from COCONUT, the library focused on COVID-19 research and other reference databases are publicly available in [29].

Johannes Kirchmair provided a succinct overview of the state-of-the-art in computational target prediction, with a focus on NPs research. He discussed the scope and limitations of 2D and 3D similarity-based methods, network-based approaches, machine learning models and docking approaches. Kirchmair talked about guidelines on how to make the best use of in silico models and understand the reliability of predictions.

Maria Sorokina presented the recently available MongoDB [30], a document-based noSQL database management system particularly suitable for storing very diverse and sparse data on NPs. Due to easy data querying and crossing, she highlighted that this database type is rapidly gaining popularity in the cheminformatics community, as more and more chemical interfaces for it are developed to enable similarity and substructure searches. She concluded her talk by showing the example of the COCONUT natural products database [31] as an illustration of a noSQL database. During the OP of Romuald Tematio Fouedjou which took place during the morning session of day 3 (dedicated to young investigators), a virtual procedure aimed at the identification of NPs with a potential affinity towards the SARS-CoV-2 main protease and spike protein based on compounds from Cameroonian medicinal plants of the Asteraceae was presented. Although the results were somewhat preliminary, the goal was to identify inhibitors that could facilitate the development of potential anti-COVID-19 drug lead compounds from Cameroonian medicinal plants.

### Hands-on sessions

Day 3 started with the parallel running of five hands-on sessions (HS). Each lasted 90 min and it was possible to switch between sessions. These sessions were received very well by the participants since they focused on direct training with selected databases and tools.

During HS01, the respective participants followed a hands-on tutorial on mining the plant specialized metabolome with help of mass spectrometry data in the Global Natural Product Social Molecular Networking (GNPS) environment (https://gnps.ucsd.edu/ProteoSAFe/static/gnps-splash.jsp) [32]. In particular, library matching and molecular networking were touched upon as well as how to study the occurrence of particular metabolites across a plant dataset when quantification information is available; for example, across genera or clades. After the main motivation to develop metabolome mining tools and a little theory, the participants got the chance to inspect the reliability of the library matches found in a recently published work studying > 70 Rhamnaceae plants from various genera and two main clades [33]. This was followed by the study of a selected number of molecular families to learn more about how molecular networking can group structurally related metabolites to propagate structural annotations within molecular families and to facilitate their joint analysis. Finally, the inclusion of quantification data into the analysis through Feature-based Molecular Networking [34] was exploited by inspecting the abundance of a number of triterpenoid and flavonoid metabolite features from across genera and clades. Even though some of these metabolites seemed highly related based on their mass fragmentation data, their abundance patterns were quite different. The hands-on session, thus, offered a quick way of reproducing and validating the results as presented in the paper [33]. The session ended with an outlook on exciting future developments in chemical compound class annotation and mass spectral similarity metrics.

**Fig. 1:**
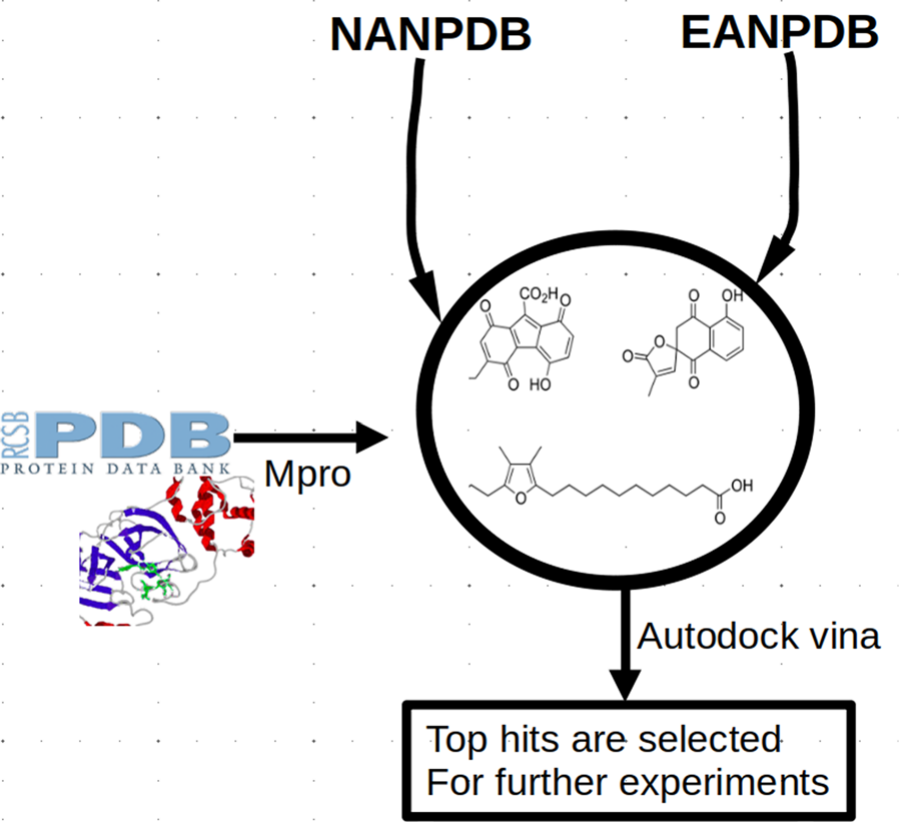
Virtual screening protocol used during the HS02 for the identification of potent natural products from African medicinal plants

The second hands-on session focused on virtual screening for the identification of bioactive SMs from NP databases, with a focus on NPs from Africa. The first presenter (Thommas M. Musyoka) introduced the South African Natural Compounds Database (SANCDB—https://sancdb.rubi.ru.ac.za/) [35], a collection of 1012 compounds derived from South African natural sources. Since its inception in 2015, the database has been used for various machine learning and in silico virtual drug screening studies with a recent study identifying several potential hits against severe acute respiratory syndrome coronavirus 2 (SARS-CoV-2). As part of a recent update, a unique feature incorporating the compound dataset analogs from two leading commercial databases (Molport—https://www.molport.com/ and Mcule—https://mcule.com/) was included. The feature not only allows users to explore a larger chemical space during screening but also enables them to seamlessly purchase compounds for their biological studies. Participants were introduced to the database by the speaker Fidele Ntie-Kang, who emphasized how they can obtain compounds in different chemical formats for both their virtual screening and biological studies. The second part of the session (approximately 20 min) focused on NPs databases originating from the regions of Northern [36] and East Africa [37] (http://african-compounds.org/anpdb/). The participants explored the web tools that enhance the search of the databases, including similarity and substructure searching for privileged scaffolds. Afterward they were introduced to the NP databases from African sources, their contents, compound classes and potential for lead compound discovery. During the last session (about 50 min), Daniel M. Shadrack introduced the participants to state-of-the-art computational techniques used in lead compound identification from electronic databases, e.g. molecular docking, and pharmacophore-based searching of the NP databases. In this section, participants were introduced to the approaches used to perform in silico screening of libraries containing natural products against the SARS-CoV-2 main protease by screening the NANPDB and EANPDB datasets against the target using the Autodock tool (see Fig. 1). The participants were also briefly introduced to other sophisticated tools like molecular dynamics and metadynamics, just on the fly, with the goal of learning how to perform virtual screening from large libraries (focusing on natural products libraries from African sources). Using this as an example, a similar approach could be used in any other project of interest, involving a different drug target.

HS03 was conducted by Yannick Djoumbou-Feunang. The first part of this session was focused on describing BioTransformer [26], an open-source software tool, and freely accessible server for the prediction of human cytochrome P450-catalyzed metabolism, human gut microbial degradation, human phase-II metabolism, human promiscuous metabolism, and environmental microbial degradation. Additionally, BioTransformer assists in metabolite identification, and metabolic pathway prediction. In the second part, an assessment of BioTransformer’s performance in the prediction of metabolism for diverse sets of molecules, including but not limited to pharmaceuticals, pesticides, and phytochemicals was presented. Overall, BioTransformer was shown to achieve moderately high precision (> 0.46), and higher recall (> 0.84) when predicting human metabolism of drugs, lipids, and phytochemicals, compared to two commercially available tools. On the other hand, the overall precision (~ 0.3) and recall (~ 0.6) achieved for the metabolism prediction of agrochemicals suggest that improvements are needed to cover more chemicals as well as biological species that are relevant to agrosciences. In the third part of this presentation, an illustration of a few examples of its application as demonstrated by various published scientific studies was provided. Furthermore, the presenter shared future perspectives for this open-source project and described how it could significantly benefit the exposure science and regulatory communities.

During the fourth HS jointly coordinated by Conrad Stork and Neann Mathai, both Ph.D. students of Johannes Kirchmair, the New E-Resource for Drug Discovery (NERDD) web service [38] was introduced to the audience. NERDD, available at https://nerdd.univie.ac.at/, provides an intuitive user interface to six cheminformatics tools: FAME3 [39] for site-of-metabolism prediction, GLORY [40] and GLORYx [41] for metabolite (structure) prediction, Hit Dexter 2.0 [42] for the prediction of frequent hitters in biological assays, NP-Scout [43] for the prediction of natural products, and Skin Doctor CP [44] for the prediction of the skin sensitization potential of small molecules. The participants were guided through the usage of each of the tools starting from the query upload to the result interpretation. For each of the tools, an exercise was given to show a range of use cases which were discussed cooperatively with the listeners.

The fifth parallel session was led by Maria Sorokina and focused on the Chemistry Development Kit (CDK) [45]. The CDK is one of the main programming toolkits for processing and analyzing chemical information. The only prerequisite for attending this workshop was some coding experience. The CDK is available as modular Java libraries, easy to use, open and free, and available to download at https://cdk.github.io/. It can also be easily integrated with both Maven and Gradle. During this hands-on, the key CDK concepts were presented, such as molecule representation and manipulation, diverse fingerprints, and molecular descriptors. Examples of the usage of CDK for natural product discovery and analysis, like the NP-likeness scorer [46] and the Sugar Removal Utility [47], were also presented. All code examples presented during this workshop were provided (Table 1). The only prerequisite for attending this workshop was some coding experience.

### Round table discussions

There were two round table discussions (RTDs). During RTD01 (following the session on bioinformatics applications which addressed issues like clustering in metabolite discovery and presentations of several web servers like SeMPI 2.0 and clustering tools OsamorSoft), several interesting questions came up, e.g., which tools would be the best to address the increasing volume of data on secondary metabolites, genomic sequences, transcriptomes, etc. To address this question, the panelists pointed to a more multidisciplinary approach that integrates the various “omics” datasets. Besides, questions were raised regarding the future of chemoinformatics for biodiversity research in Brazil, as the morning speaker (Marilia Valli) had mentioned that many of the biodiversity hotspots were not well represented in their collected NuBBE database. To address this, the speaker mentioned that although there is still a majority of species in Brazil to be studied, the published works are being added to NuBBE Database. During the RTD02 chaired by early career researchers, Serge A. T. Fobofou first of all appreciated the organizers for the effort put together to bring together top researchers to such a workshop and for making the workshop freely accessible to all, thus saving a huge financial cost. Feedback from the parallel session was received during the first part of the round table discussion that followed that session (RTD02). In general, the participants agreed with the current format of online presentations, but would have preferred to have a workshop with physical presence. The chair further invited participants to ask specific questions to the individual speakers before making suggestions for the future. The questions from the participants pointed to the fact that the workshop contents were received with a lot of enthusiasm, suggesting that a compendium of computational tools that were introduced during the workshop be compiled and made available to all participants. This has been made available in Table 1.

## Conclusions

SMs play important roles in agricultural, cosmetic and pharmaceutical industries and the mastery of the use of computational methodologies for their investigation is urgently needed in the scientific community. The importance of this workshop is that it was able to bring together 195 registered participants and 24 experts from around the world, and that the participants would benefit from 3 days of intensive training free of cost. Basically, the attendees were drilled on state-of-the-art in silico methodologies, algorithms and tools that could be useful in the rapid discovery of NPs for small molecule drug discovery. A compendium of in silico tools to enhance NP dereplication, lead discovery and de-novo design was made available to all participants. The workshop lectures and materials shown and worked with during the hands-on sessions have been made available for download from the workshop website. Participants from low income countries who, otherwise, would not be able to attend such a high profiled meeting could do so at no cost and have access to the software and servers that would enhance their discovery of drugs from NP-based leads. It is hoped that these would serve as a foundation for early-career researchers working or starting off their studies in this field. Besides, the responses from the feedback survey will serve the purpose of improving subsequent similar events. The workshop ended with a round table discussion led by early-career researchers to summarize the key events of the meeting.

## Supplementary Information

**Additional file 1. **Workshop statistics.

**Additional file 2. **CAiSMD Feedback form.

## Abbreviations

ANPDB: African Natural Products Database
BGC: Biosynthetic gene cluster
CAS: Chemical Abstracts Service
CDK: Chemistry Development Kit
GNPS: The Global Natural Product Social Molecular Networking
HS: Hands-on session
KL: Keynote lecture
MAGMa: An online application for the automatic chemical annotation of accurate multistage MS^n^ spectral data
MS2LDA: A tool that decomposes molecular fragmentation data derived from large metabolomics experiments into annotated Mass2Motifs
NERDD: New E-Resource for Drug Discovery
NP: Natural product
NRPS: Nonribosomal Peptide-Synthetase
nsSNVs: Nonsynonymous Single Nucleotide Variations
OP: Oral presentation
PKS: Polyketide Synthase
RTD: Round table discussion
SANCDB: South African Natural Compounds Database
