# Supplementary material for: Computational Applications in Secondary Metabolite Discovery (CAiSMD): an online workshop
Source: J Cheminform. 2021 Sep 6;13:64. doi: 10.1186/s13321-021-00546-8 (PMC8419829; doi:10.1186/s13321-021-00546-8)
Supplement: Supplementary file 1 — Additional file 1. Workshop statistics. [file 13321_2021_546_MOESM1_ESM.docx]

Meeting Report

**Computational Applications in Secondary Metabolite Discovery (CAiSMD): An online workshop**

Fidele Ntie-Kang^1,2,3*^, Kiran K. Telukunta^4^, Serge A. T. Fobofou^5^, Victor C. Osamor^6^, Samuel A. Egieyeh^7,8^, Marilia Valli^9^, Yannick Djoumbou Feunang^10^, Maria Sorokina^11^, Conrad Stork^12^, Neann Mathai^13^, Paul Zierep^14^, Ana L. Chávez-Hernández^15^, Miquel Duran-Frigola^16,17^, Smith B. Babiaka^1^ , Romuald Tematio Fouedjou^18^, Donatus B. Eni^1^, Simeon Akame^19^, Augustine B. Arreyetta-Bawak^20^, Oyere T. Ebob^1^, Jonathan A. Metuge^20^, Boris D. Bekono^21^, Mustafa A. Isa^22^, Raphael Onuku^23^, Daniel M. Shadrack^24^, Thommas M. Musyoka^25^, Vaishali M. Patil^26^, Justin J. J. van der Hooft^27^, Vanderlan da Silva Bolzani^9^, José L. Medina-Franco^15^, Johannes Kirchmair^28^, Tilmann Weber^29^, Özlem Tastan Bishop^25^, Marnix H. Medema^27^, Ludger A. Wessjohann^30,31^ and Jutta Ludwig-Müller^3*^

^1^ Department of Chemistry, University of Buea, P. O. Box 63 Buea, Cameroon

^2^ Institute of Pharmacy, Martin-Luther University of Halle-Wittenberg, Kurt-Mothes-Str. 3, 06120 Halle, Germany

^3^ Institute of Botany, Technische Universität Dresden, Zellescher Weg 20b, 01062 Dresden, Germany

^4^ Tarunavadaanenasaha Muktbharatonnayana Samstha Foundation, Hyderabad, India

^5^ Institute of Pharmaceutical Biology, Technische Universität Braunschweig, Mendelssohnstrasse 1, 38106 Braunschweig, Germany

^6^ Department of Computer and Information Sciences, Covenant University, Ota, Nigeria

^7^ School of Pharmacy, University of the Western Cape, Cape Town, 7535 South Africa ^8^ South African Medical Research Council Bioinformatics Unit, South African National Bioinformatics Institute, University of the Western Cape, Cape Town 7535, South Africa

^9^ Nuclei of Bioassays, Biosynthesis and Ecophysiology of Natural Products (NuBBE), Department of Organic Chemistry, Institute of Chemistry, Sao Paulo State University – UNESP, Araraquara, Brazil

^10^ Corteva Agriscience, Indianapolis, Indiana, USA ^11^ Institute for Inorganic and Analytical Chemistry, Friedrich Schiller University, Jena, Germany ^12^ Center for Bioinformatics, Universität Hamburg, 20146 Hamburg, Germany

^13^ Department of Chemistry and Computational Biology Unit (CBU), University of Bergen, N-5020 Bergen, Norway ^14^ Pharmaceutical Bioinformatics, Albert-Ludwigs-University Freiburg, Germany

^15^ DIFACQUIM Research Group, Department of Pharmacy, School of Chemistry, Universidad Nacional Autónoma de México, México City, México

^16^ Ersilia Open Source Initiative, Cambridge, UK

^17^ Joint IRB-BSC-CRG Programme in Computational Biology, Institute for Research in Biomedicine (IRB Barcelona), The Barcelona Institute of Science and Technology, Barcelona, Catalonia, Spain

^18^ Department of Chemistry, University of Dschang, Dschang, Cameroon

^19^ Department of Immunology of Public Health, School of Health Sciences, Catholic University of Central Africa, BP 7871, Yaoundé, Cameroon ^20^ Department of Biochemistry and Molecular Biology, University of Buea, P. O. Box 63 Buea, Cameroon

^21^ Department of Physics, Ecole Normale Supérieure, University of Yaoundé I, BP. 47, Yaoundé, Cameroon ^22^ Bioinformatics and Computational Biology Lab, Department of Microbiology, Faculty of Sciences, University of Maiduguri, P.M.B. 1069, Borno State, Nigeria

^23^ Department of Pharmaceutical and Medicinal Chemistry, Faculty of Pharmaceutical Sciences, University of Nigeria Nsukka, Nsukka, Nigeria ^24^ Department of Chemistry, St. John’s University of Tanzania, P. O. Box 47 Dodoma, Tanzania ^25^ Research Unit in Bioinformatics (RUBi), Department of Biochemistry and Microbiology, Rhodes University, Makhanda, 6140, South Africa

^26^ Computer Aided Drug Design Lab, KIET Group of Institutions, Delhi-NCR, Ghaziabad-201206, India  ^27^ Bioinformatics Group, Wageningen University, Wageningen, the Netherlands ^28^ Department of Pharmaceutical Sciences, Division of Pharmaceutical Chemistry, University of Vienna, 1090 Vienna, Austria

^29^ The Novo Nordisk Foundation Center for Biosustainability, Technical University of Denmark, Kgs. Lyngby, Denmark ^30^ Department of Bioorganic Chemistry, Leibniz Institute of Plant Biochemistry (IPB), Weinberg 3, D-06120 Halle (Saale), Germany

^31^ German Centre for Integrative Biodiversity Research (iDiv), Puschstraße 4, 04103 Leipzig, Germany

*****Correspondence: [fidele.ntie-kang@ubuea.cm](mailto:fidele.ntie-kang@ubuea.cm) (FN-K) and [jutta.ludwig-mueller@tu-dresden.de](mailto:jutta.ludwig-mueller@tu-dresden.de) (JL-M)

**ADDITIONAL FILE 1**

**Workshop statistics**

It was generally observed that apart from spreading the information among colleagues (63% of those who participated in the post-workshop workshop stated they were informed of the workshop by a colleague, see the feedback summary in the Supplementary Data), social media (e.g. Twitter, LinkedIn, etc.) was suggested. In addition, an anonymous online survey was conducted during which all participants mentioned that it was quite easy to complete the online registration form. The average rating of the speakers was 8.62/10.00 and 8.45/10.00 for the workshop contents. About 96% of the participants could easily find the zoom link and 97% of the participants found the book of abstracts useful. Besides, 90% found the break periods acceptable, while 6% found them too long and 4% found them too short. The overall usefulness of the workshop was rated at 8.79/10.00 by those who participated in the survey. 99% would recommend this workshop to a colleague, although 39% found the timetable too tight to follow. Regarding the website contents, the accessibility of its contents was rated averagely as 8.37/10.00, while 97% of the participants in the survey requested a certificate of participation. Regarding which aspects of the workshop (see Supplementary Data 2) would be regarded as the most important for the participants, it was shown, from the polls, that 44% voted for the oral presentations, followed by the hands-on sessions (39%), the keynote lectures (35%), the round table discussions (20%) and last of all the interactive session (1%). More time was, therefore, requested for interaction subsequently.

During the post-workshop survey the feedback showed that a number of points should be improved in subsequent editions:

1. There was no session during which at least half of the 195 officially registered participants attended. Some comments during the feedback showed that some participants were either held back by other scientific activities, were following from geographical locations with substantial time differences from CET, or several participants followed the workshop as a group (using the same computer) from the same venue and had to be logged in as a single user.
2. Many participants would have preferred the hands-on sessions not to be held in parallel. Some speakers of the parallel sessions had wished to follow the other sessions as well. This emphasized the need to avoid running parallel hands-on sessions in subsequent editions of the workshop. This would also avoid the discrepancy in the number of participants in groups of hands-on sessions.
3. More than half of the participants indicated that they had preferred more hands-on sessions and fewer lectures and presentations. Others mentioned that 15 minutes lectures were too short. They would have preferred 20 to 25 minutes lectures to allow discussion time with participants.
4. While many participants reported that they did not consult the workshop website prior to the beginning of the meeting, 90% of those who consulted the website commended its contents.
5. Some website users suggested the organizers improve accessibility by including better links to contact speakers, e.g., advertise through social media (Tweeter at least).
6. Harmonizing the time for participants from the continents of Asia, North and South America to follow the workshop more conveniently proved challenging. It was suggested to get permission from speakers to record the sessions for participants in different time zones to later follow at a more convenient time.
7. Participants also suggested that the organizers plan additional time for Q&A sessions during hands-on sessions in order to give further in-depth description of the chemoinformatic tools and create sessions for practical tutorials with participants.
8. Some participants suggested that it would be helpful to add information about the various links available on the website. These have now been published in the supplementary data of this report and included in the workshop website.
9. More time could be dedicated to hands-on sessions to ease participants' understanding. Moreover, since some of the participants are beginners, it was also suggested to include some preliminary introductory or foundational courses, not just research results.
10. More resources on NP research should be uploaded and also the profiles of the resource person should be made available for future reference and correspondence. These are currently available on the website and the updated book of abstracts in the 'downloads' section of the workshop website.
11. More workshops should be organized but the schedule should be planned well in advance to improve planning. Slides could be changed in order for everyone to see in detail what is on slides. The workshop slides are now available on the workshop website for download.
12. The workshop links to the zoom sessions should be shared well in advance, with a reminder 10 minutes before starting the session. Some suggestions were to use the "Go To Meeting" tool while others would prefer that the workshop be recorded and made live on YouTube.
