# Supplementary material for: Computational Applications in Secondary Metabolite Discovery (CAiSMD): an online workshop
Source: J Cheminform. 2021 Sep 6;13:64. doi: 10.1186/s13321-021-00546-8 (PMC8419829; doi:10.1186/s13321-021-00546-8)
Supplement: Supplementary file 2 — Additional file 2. CAiSMD Feedback form. [file 13321_2021_546_MOESM2_ESM.pdf]

CAiSMD Feedback form

CAiSMD Feedback form

How did you get to know about this workshop?

67 Responses- 3 Empty

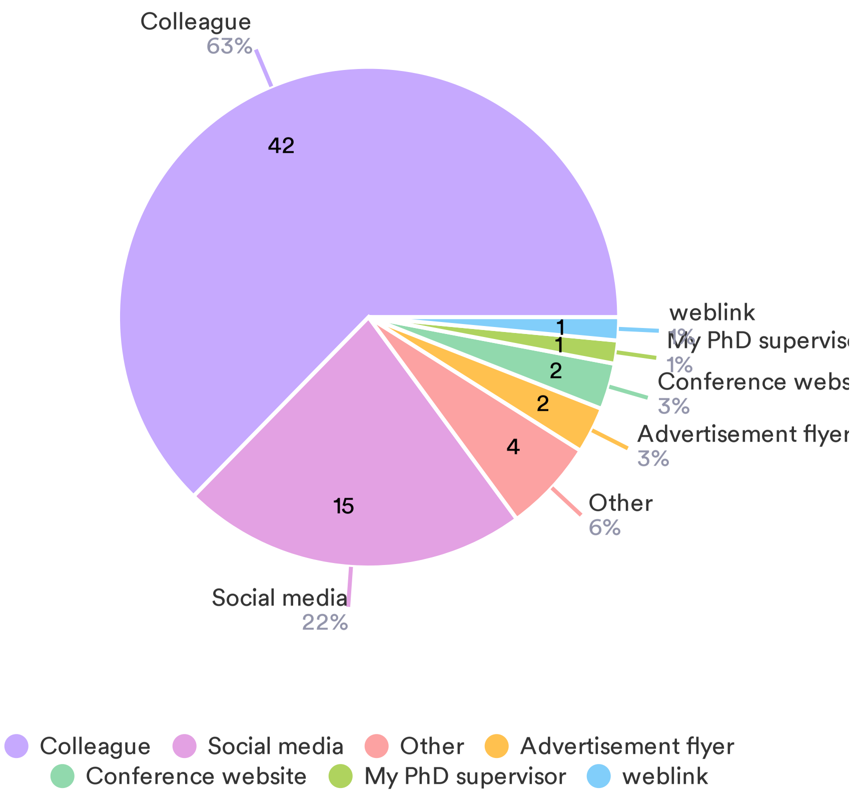

Was it easy to register online?

69 Responses- 1 Empty

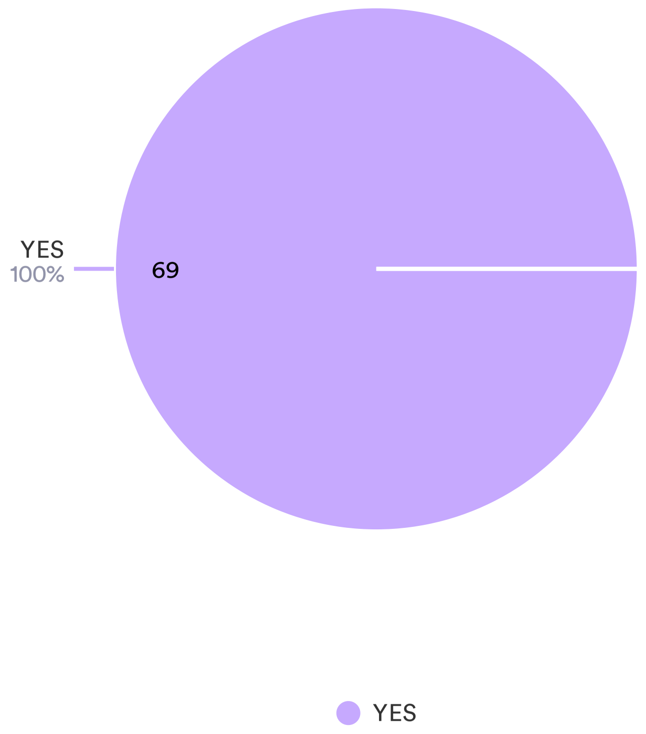

How did you find the speakers?

69 Responses- 1 Empty

8.62
Avg. Response

69
Responses

How did you find the contents of the workshop?

69 Responses- 1 Empty

8.45
Avg. Response

69
Responses

CAiSMD Feedback form

Did you find the zoom link easily available?

68 Responses- 2 Empty

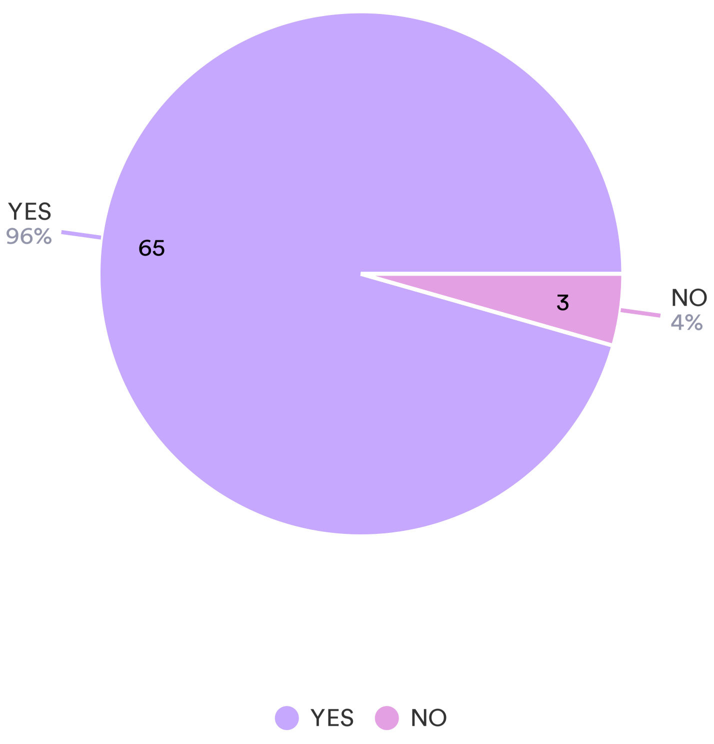

Which meeting tool you would have preferred?

3 Responses- 67 Empty

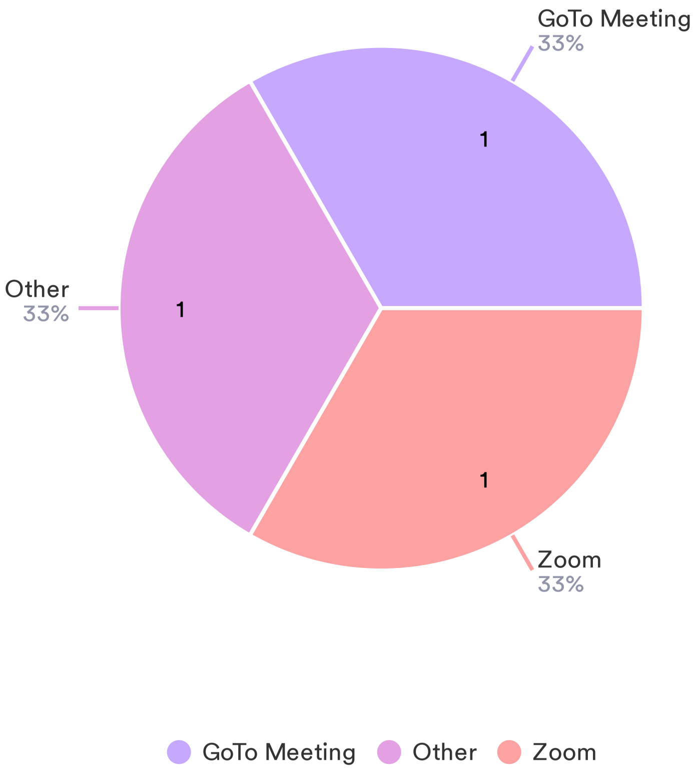

CAiSMD Feedback form

Did you find the book of abstracts useful?

69 Responses- 1 Empty

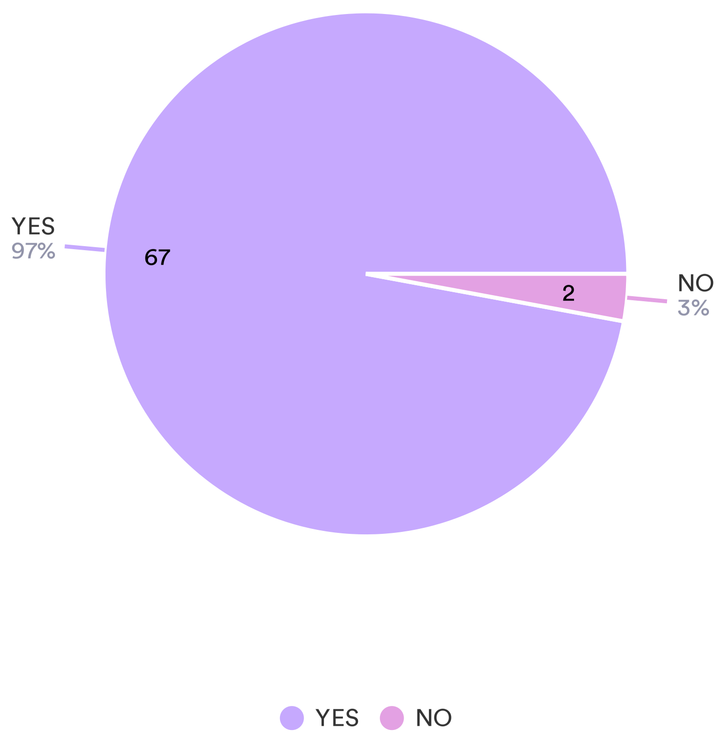

What did you find most useful?

163 Responses- 1 Empty

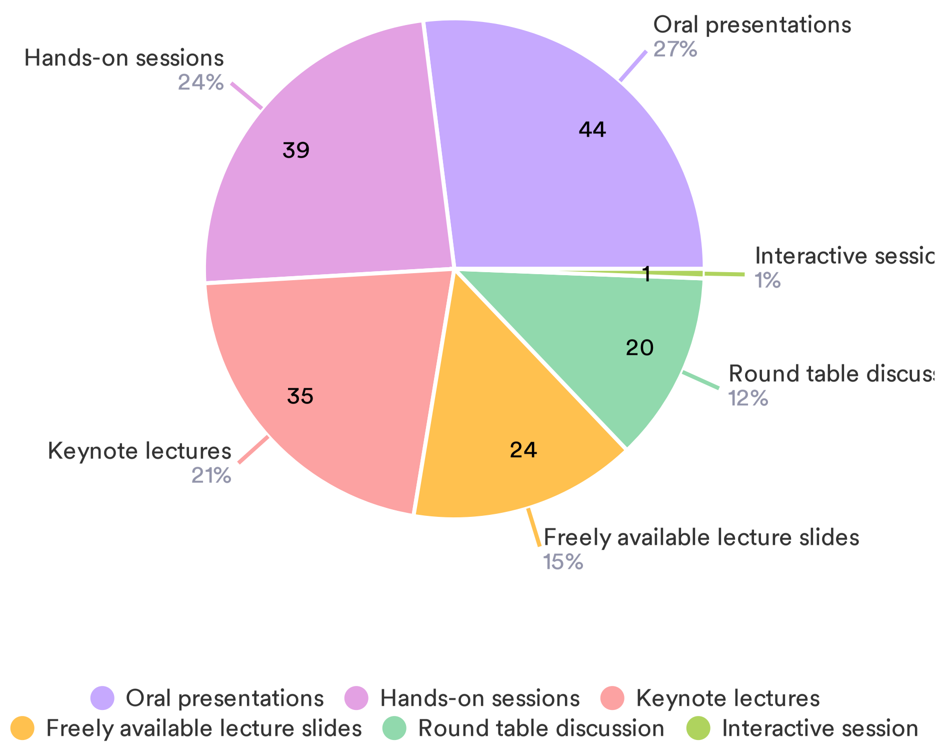

How did you find the break periods?

69 Responses- 1 Empty

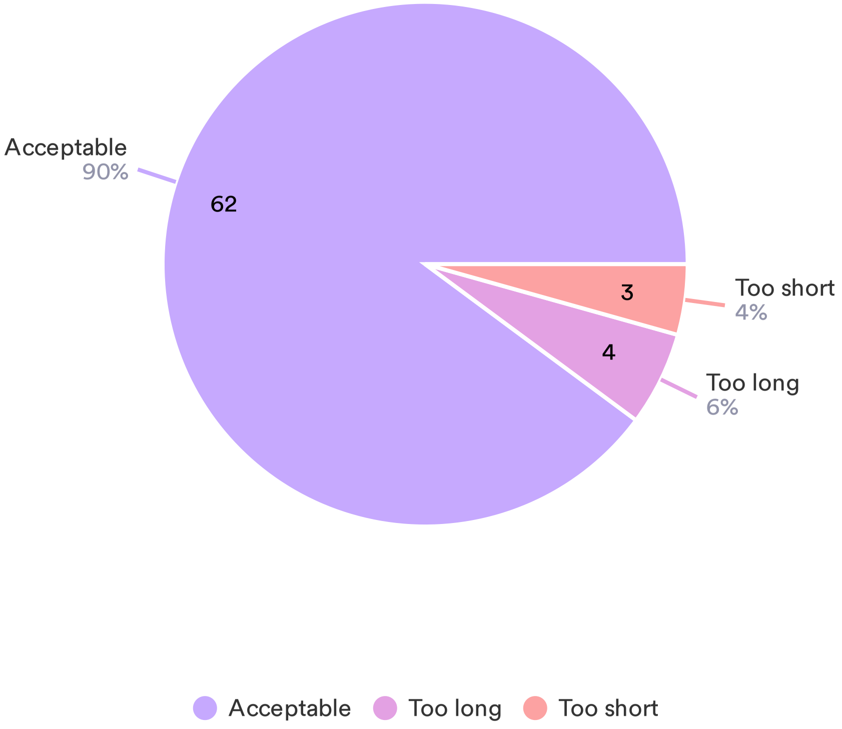

How useful did you find this workshop?

68 Responses- 2 Empty

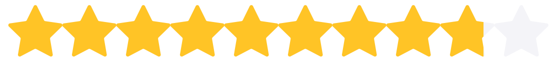

8.79
Avg. Response

68
Responses

Would you recommend this workshop to a colleague?

68 Responses- 2 Empty

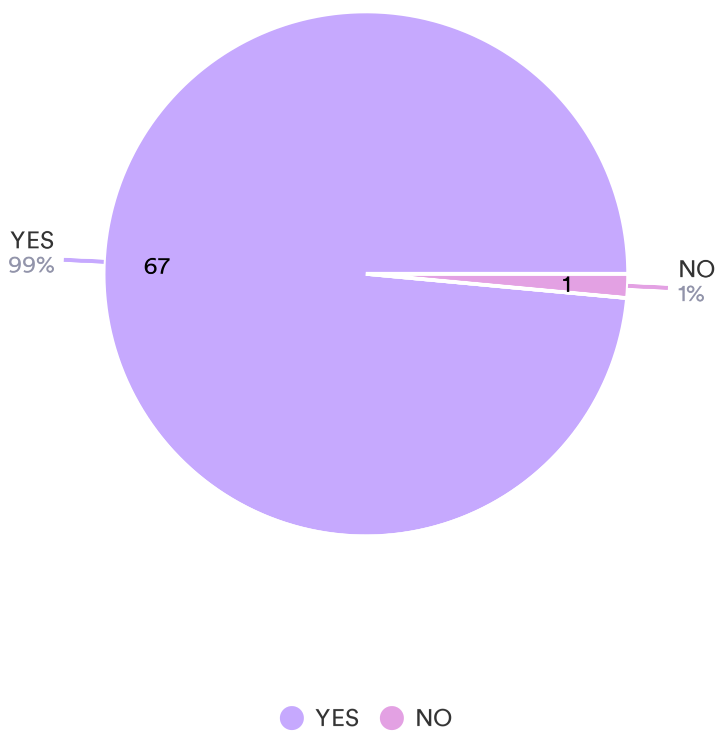

Was the timetable too tight to follow?

69 Responses- 1 Empty

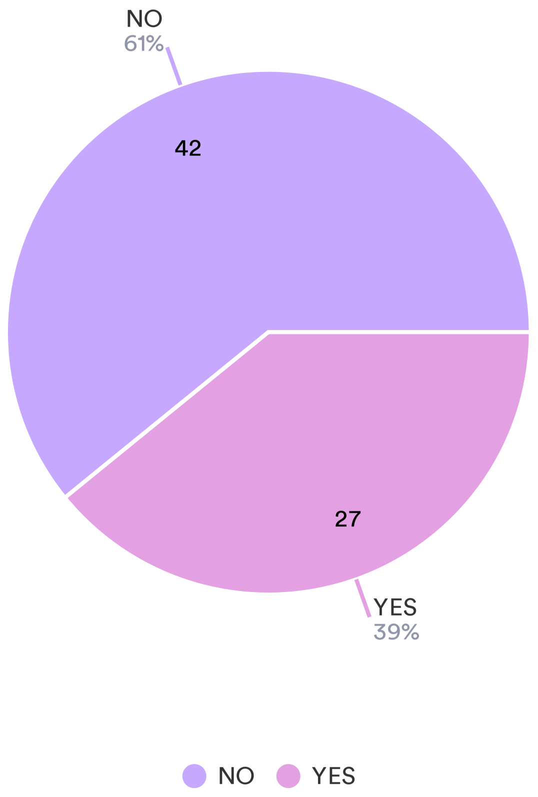

How easy was accessing website?

68 Responses- 2 Empty

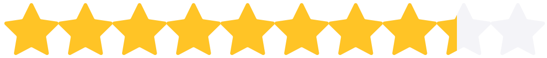

8.37
Avg. Response

68
Responses

Did you find all information you want in website?

69 Responses- 1 Empty

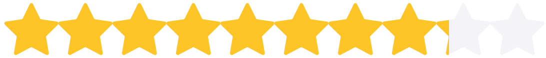

8.23
Avg. Response

69
Responses

Do you have any suggestions to improve the website?

38 Responses- 32 Empty

Data	Responses
No	5
NA	5
No suggestions	1
Sorry I don't have.	1
No suggestion	1
No, I do not.	1
I believe there is still room for improvement but would like to get to you if I have any.	1
Not at all	1
More resources on NP research should be uploaded and also profile of resource person should be made available for future	1

Do you have any other suggestions for improving the workshop?

44 Responses- 26 Empty

Data	Responses
No	6
NA	3
None	2
The organizers could give more time for the hands-on session	1
the zoom meeting links should be clearly marked out for each session at the different session	1
First of all thank you very much organizers for this workshop, congratulations !!! Basically, I have 2 suggestions; 1- More time for hands-on sessions because it gives more time for exchanges hence provides higher opportunities for learning. 2- Though difficult, I firmly think that a face to face workshop would be more appreciated. So bringing people together in one place for me is my second suggestion. This is because it is difficult to respect time with our different geographical time zones.	1
